# G9a-dependent histone methylation can be induced in G1 phase of cell cycle

**DOI:** 10.1038/s41598-018-37507-5

**Published:** 2019-01-30

**Authors:** Mikiko Fukuda, Asako Sakaue-Sawano, Chikako Shimura, Makoto Tachibana, Atsushi Miyawaki, Yoichi Shinkai

**Affiliations:** 1 Cellular Memory Laboratory, RIKEN Cluster for Pioneering Research, 2-1 Hirosawa, Wako, Saitama, 351-0198 Japan; 2 Laboratory for Cell Function Dynamics, RIKEN Center for Brain Science, 2-1 Hirosawa, Wako, Saitama, 351-0198 Japan; 3 Institute of Advanced Medical Sciences, Tokushima University, 3-18-15 Kuramoto-cho, Tokushima, 770-8503 Japan; 4 Experimental Research Center for Infectious Diseases, Institute for Virus Research, Kyoto University, 53 Shogoin, Kawara-cho, Sakyo-ku, Kyoto, 606-8597 Japan

## Abstract

Epigenetic information (epigenome) on chromatin is crucial for the determination of cellular identity and for the expression of cell type-specific biological functions. The cell type-specific epigenome is maintained beyond replication and cell division. Nucleosomes of chromatin just after DNA replication are a mixture of old histones with the parental epigenome and newly synthesized histones without such information. The diluted epigenome is mostly restored within one cell cycle using the epigenome on the parental DNA and nucleosomes as replication templates. However, many important questions about the epigenome replication process remain to be clarified. In this study, we investigated the model system comprising of dimethylated histone H3 lysine 9 (H3K9me2) and its regulation by the lysine methyltransferase G9a. Using this epigenome model system, we addressed whether H3K9me2 can be induced in specific cell cycle stages, especially G1. Using cell cycle-specific degrons, we achieved G1 or late G1-to M phases specific accumulation of exogenous G9a in G9a deficient cells. Importantly, global levels of H3K9me2 were significantly recovered by both cell types. These data indicate that H3K9me2 may be plastic and inducible, even in the long-living, terminally-differentiated, post-mitotic, G0-G1 cell population *in vivo*. This knowledge is valuable in designing epigenome-manipulation-based treatments for diseases.

## Introduction

The human body consists of hundreds of different cell types. Their unique cellular nature is maintained beyond cell division once cellular identities have been established. Since the epigenome controls gene functions, a cell type-specific epigenome must be inherited by daughter cells to maintain their specific gene functions. DNA methylation is faithfully maintained throughout cell division by the DNA methyltransferase DNMT1 pathway. The pattern of histone post-translational modifications (PTMs), especially lysine methylations, is also maintained beyond cell division. However, the molecular details of the inheritance of histone methylation are limited.

The recently developed stable isotope labeling with amino acids in cell culture (SILAC)-based quantitative mass spectrometry technology has clarified the dynamics of histone PTMs. Histone lysine di- and tri-methylation are diluted twofold upon DNA replication, but most of them are restored within one cell cycle^1–6^. However, many important problems remain unclear, such as the mode of recovery of different histone PTMs and their cell cycle-specific regulatory roles.

To address above these unknowns concerning epigenetic inheritance, we used the lysine methyltransferase G9a (also known as EHMT2) and H3K9me2 regulated by the G9a^7^ as a model system to address how G9a-mediated H3K9me2 is regulated across the different cell cycle stages. Although G9a and the G9a-related molecule GLP (also known as EHMT1) individually display H3K9 methyltransferase activity *in vitro*, G9a and GLP usually form a heteromeric complex that is essential for their H3K9 methylation activity *in vivo*. Furthermore, enzymatic activity of G9a in the G9a/GLP complex has a dominant role in the H3K9 methylation^8^. In the histone methylation assay, G9a and GLP prefer the core-histone as substrate and not the nucleosomal histone^9^. Neighboring nucleosomes containing H3K9me1 or me2 are able to stimulate their methylation catalysis of the nucleosomal histone^10^. This stimulation activity works in cis and requires the methylated H3K9 binding activities of ankyrin repeat domains of GLP and G9a^10,11^. This suggests that parental nucleosomes containing H3K9 methylation may play crucial role in recovery of G9a/GLP-mediated H3K9 methylation landscape beyond the cell division *in vivo*.

H3K9me1 and H3K9me2 is diluted twofold upon DNA replication but is quickly restored during the G2 phase and is almost completely recovered before the next DNA replication. However, the dynamics of G9a/GLP-mediated H3K9 methylation in cell cycle stages have not been well examined. To address how G9a/GLP-mediated H3K9 methylation functions in different cell cycle stages, we tried to establish cells expressing G9a only during the G1 or the out-of-G1 phases. The degrons of the cell cycle-specific proteins, Cdt1 and Geminin, which are absent during the S, G2, and M (S/G2/M) phases, and the G1 phase, respectively, are the basis of the fluorescent ubiquitination-based cell cycle indicator (Fucci) system^12^, which is specific for G1 and out-of-G1 phases. Using this degron system, we achieved in making cells that express G9a only during the G1 or late G1-to-M phases.

## Results

### Establishment of cell line expressing G9a restricted to G1 or out-of-G1 cell cycle

**Figure 1:**
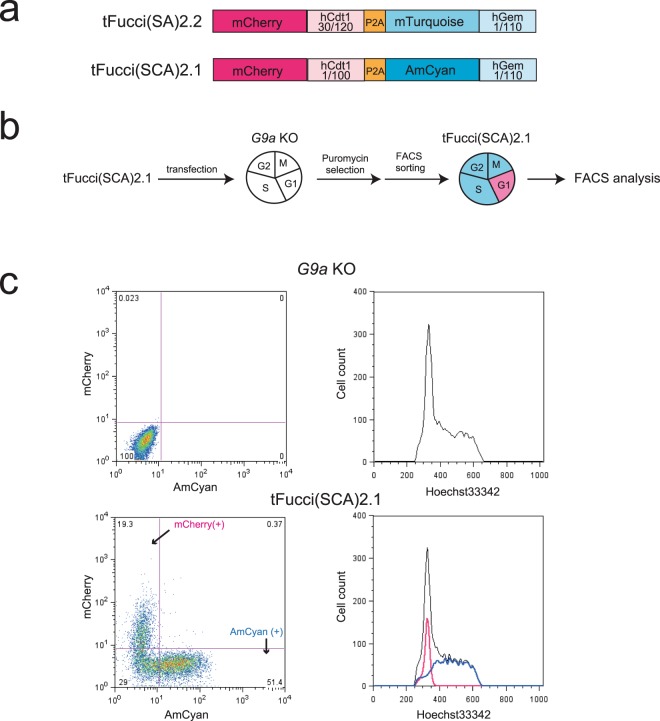
Establishment of *G9a* KO iMEFs expressing tFucci(SCA)2.1. (**a**) Construction of tFucci(SCA)2.1. The modification of the tFucci(SA)2.2 system comprised mCherry-hCdt1(1/100), P2A, and AmCyan-hGeminin(1/110). (**b**) Strategy for the establishment of *G9a* KO iMEFs expressing tFucci(SCA)2.1. (**c**) Fluorescence-activated cell sorting (FACS) analysis of the expression of mCheery and AmCyan (left panels) and DNA contents (right panels). Black line: total cells, blue line: AmCyan (+) cells, red line: mCherry (+) cells.

To construct cells that express G9a only during the G1 or out-of-G1 phases, we first introduced tFucci(SCA)2.1, which is a modified version of tFucci(SA)2.2^13^ constructed from hCdt1(1/100) fused with mCherry and hGeminin(1/110) fused with AmCyan (Fig. 1a) into *G9a* knockout (KO) cells of immortalized mouse embryonic fibroblast (iMEFs) (Fig. S1a). tFucci(SCA)2.1 allows for the improved expression of more restricted G1 phase of mCherry by replacement of hCdt1(30/120) with hCdt1(1/100). Furthermore, in tFucci(SA)2.2, mTurquoise-hGeminin(1/110) is used for out-of-G1 phase monitoring, although it is possible that this vector could recombine with any vector containing the *mVenus* gene inside the cells, because of the high sequence similarity between mTurquoise and mVenus. Therefore, mTurquoise was replaced with AmCyan in tFucci(SCA)2.1. After the transfection of tFucci(SCA)2.1 into *G9a* KO iMEFs, the cells were selected with puromycin, and AmCyan single positive cells were sorted using fluorescence-activated cell sorting (FACS) (Fig. 1b). The sorted iMEFs were grown and further characterized by FACS with Hoechst 33342 staining. As expected, the iMEFs transfected with tFucci(SCA)2.1 detected the AmCyan in the S/G2/M phases, but not in the G1 phase, and mCherry was detected only in the G1 phase of the cell cycle (Fig. 1c).

**Figure 2:**
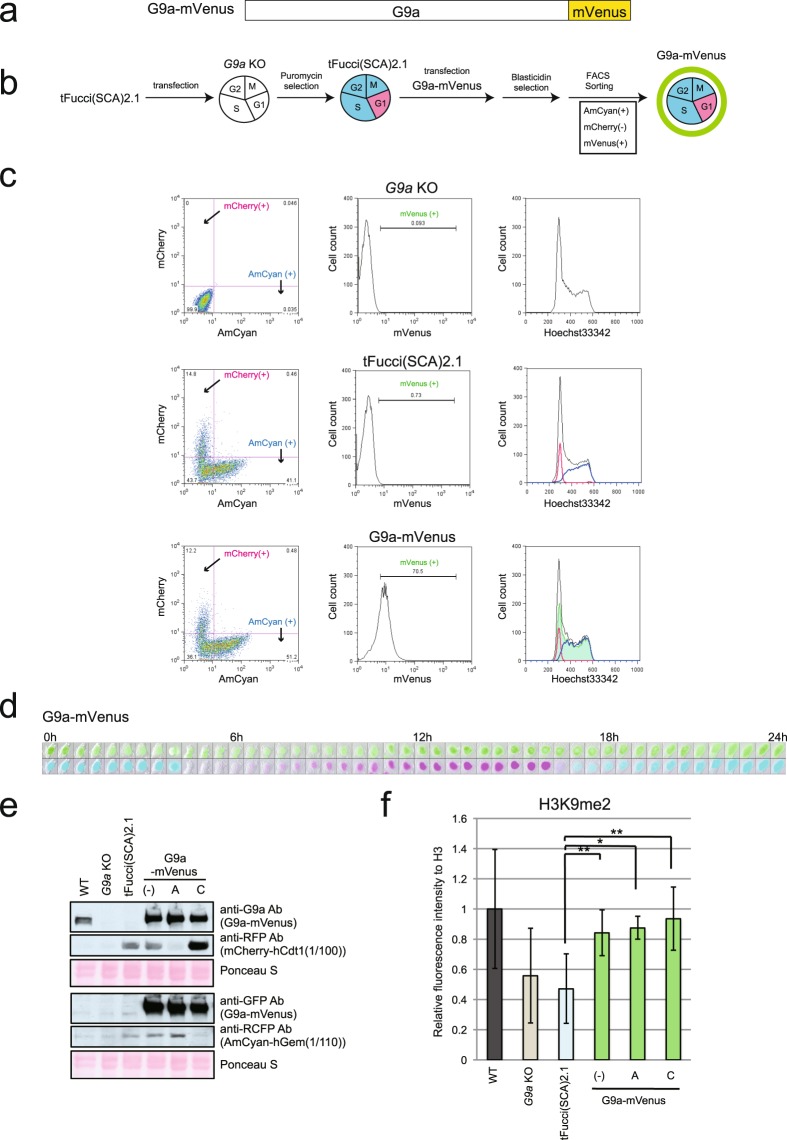
Establishment of *G9a* KO iMEFs expressing G9a-mVenus. (**a**) Construction of G9a-mVenus. G9a was fused to mVenus at the C-terminus. (**b**) Strategy for the establishment of the *G9a* KO iMEFs expressing G9a-mVenus. (**c**) FACS analysis of the expression of mCheery and AmCyan (left panels), mVenus (middle panels), and DNA contents (right panels). Black line: total cells, blue line: AmCyan (+) cells, red line: mCherry (+) cells and green line: mVenus(+). (**d**) The cell line expressing G9a-mVenus was live imaged by LCV110. The images were excerpts taken during the first 24 h. mVenus (upper panels), and AmCyan and mCherry (lower panels) are showed in combination in bright field images. They were photographed every 30 min. e) G9a-mVenus protein was detected using anti-G9a antibody and anti-GFP antibody by western blot. mCherry and AmCyan also was detected using to confirmation of the sorting specificity. (−): total cells, A: AmCyan (+) sorted cells, C: mCherry (+) sorted cells. (**f**) H3K9me2 level was determined by western blot using Odyssey CLs. The means of relative fluorescence intensity to H3 is shown in the graphs. N = 3, independent experiments. Original images are shown in Fig. S3. Error bars indicate ± SD *p < 0.05 and **p < 0.01 by Student’s t-test. Compared to WT, *G9a* KO and tFucci(SCA)2.1 showed statistically significant differences (p < 0.05).

Before trying to establish cell cycle-specific G9a expressing cells, we examined endogenous G9a protein level in different cell cycle in iMEFs. As shown in Fig. S2, G9a cellular content was constitutively maintained throughout the entire cell cycle and did not decrease in the G1 phase. We also introduced the constitutively expressing G9a-mVenus construct (Fig. 2a) into *G9a* KO iMEFs with tFucci(SCA)2.1 and examined the impact of this G9a-mVenus expression on H3K9me2. After selecting for vector transfection using blasticidin, AmCyan and mVenus double-positive cells were sorted by FACS (Fig. 2b). The sorted cells were further analyzed by FACS with Hoechst 33342 staining (Fig. 2c), live fluorescent imaging of independent cells was carried out (Fig. 2d), and western blot analysis of the sorted AmCyan or mCherry-positive populations was performed (Fig. 2e). These results demonstrated that, as expected, G9a-mVenus was expressed in cell nuclei in both G1 and out-of-G1 cell cycles. The sorted G1 and out-of-G1 cell cycle phase populations were then characterized for their H3K9me2 status (Figs 2f and S3). Western blot analysis clearly demonstrated that the level of H3K9me2 was significantly recovered in *G9a* KO iMEFs expressing G9a-mVenus in both G1 and out-of-G1 phase populations.

**Figure 3:**
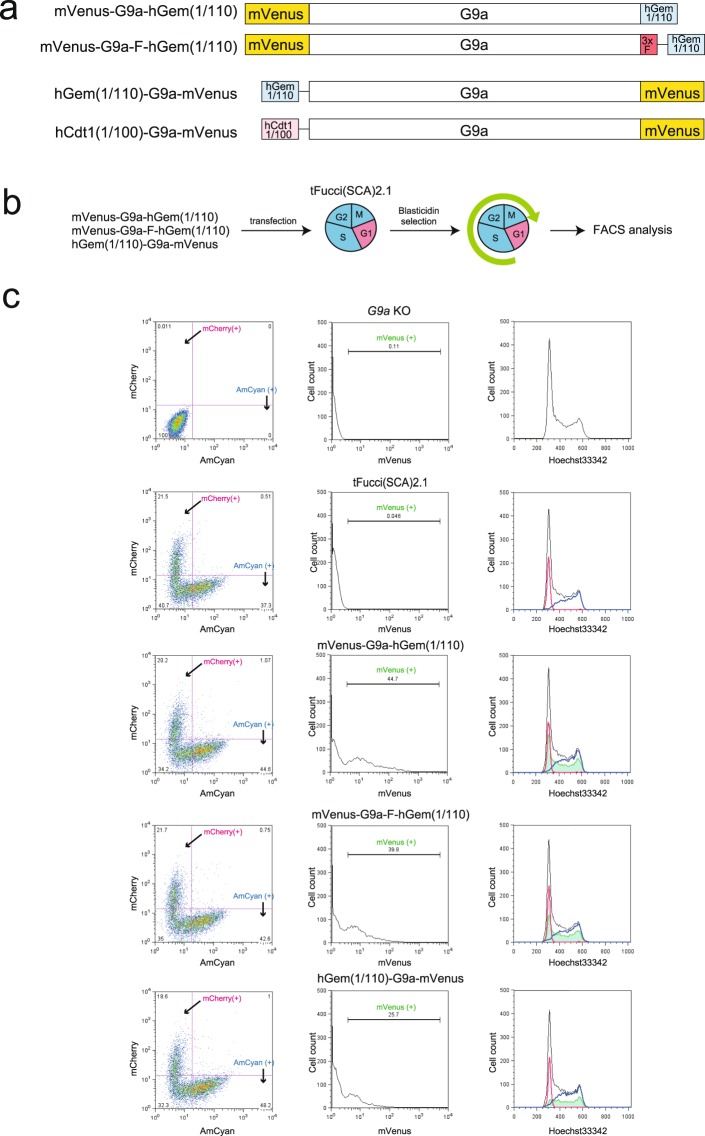
Examination of hGeminin degron conjugated G9a-mVenus degradation. (**a**) Construction of the G9a expression vectors using the Fucci system. G9a was fused hGeminin(1/110) or hCdt1(1/100) and mVenus. Additionally mVenus-G9a-F-hGem(1/110) contained 3 × Flag to the C-terminus of G9a. Furthermore, mVenus-G9a-F-hGem(1/110), hGem(1/110)-G9a-mVenus, and hCdt1(1/100)-G9a-mVenus contained linker DNA coupler1 between G9a and Gem or Cdt1 (thin line). (**b**) Strategy of the tFucci(SCA)2.1 analysis of iMEFs transiently expressing hGeminin(1/110) fused G9a vectors. (**c**) FACS analysis of the expression of mCheery and AmCyan (left panels), mVenus (middle panels), and DNA contents (right panels). Black line: total cells, blue line: AmCyan (+) cells, red line: mCherry (+) cells, and green line: mVenus(+).

Subsequently, we aimed to establish the cell lines where G9a is specifically expressed in G1 or out-of-G1 cell cycles. For this purpose, the following fusion constructs were prepared: mVenus-G9a-hGeminin(1/110) (also termed mVenus-G9a-hGem(1/110)); mVenus-G9a-3xFlag-coupler1-hGeminin(1/110) (also termed mVenus-G9a-F-hGem(1/110)); and hGeminin(1/110)-coupler1-G9a-mVenus (also termed hGem(1/110)-G9a-mVenus) (Fig. 3a). Coupler1 is the linker DNA encoding glycine-rich sequences, which allows efficient target protein degradation by the conjugated degron-induced proteasome-mediated proteolysis^14^. These vectors were transfected into *G9a* KO iMEFs expressing tFucci(SCA)2.1, selected with blasticidin, and analyzed using FACS with Hoechst 33342 staining (Fig. 3b). The rational of the assay is that fluorescent signals of mVenus should be detected in the S/G2/M phases, but not in the G1 phase of the cell cycle, if the amount of mVenus-G9a fusion protein is controlled by the conjugated hGeminin(1/110), as in Fucci. However, mVenus-G9a-hGem(1/110) and mVenus-G9a-F-hGem(1/110) were detected all the in phases. On the other hand, if hGem(1/110)-G9a-mVenus was used, mVenus signals were mostly not overlapped with the mCherry signals as were observed for the mVenus-G9a-hGem(1/110) constructs but still induced before starting S phase (Fig. 3c right panels), suggesting that hGem(1/110)-G9a-mVenus is significantly diminished in early G1 phase but appears from late G1 to M phase. Since we found that conjugation of the hGeminin degron to the 5′ end of G9a participates more in regulating the cell cycle (S/G2/M phases) G9a (G9a-mVenus) expression, we constructed hCdt1(1/100)-coupler1-G9a-mVenus (also termed hCdt1(1/100)-G9a-mVenus) for G1 cell cycle-specific G9a expression (Fig. 3a).

**Figure 4:**
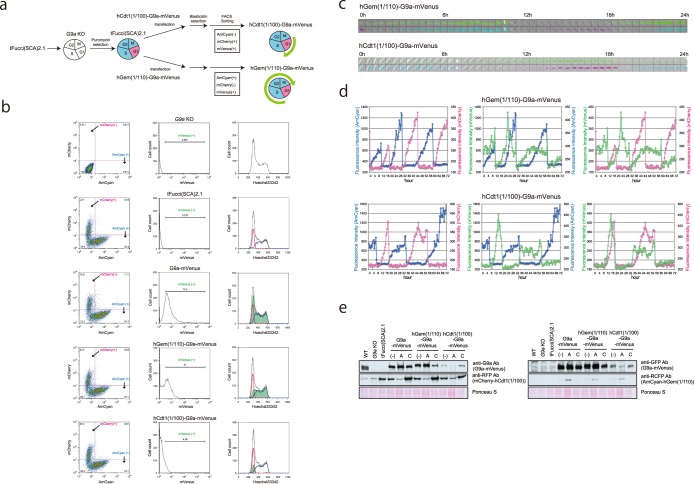
Establishment of *G9a* KO iMEFs expressing G9a-mVenus that was degraded in a specific phase of the cell cycle. (**a**) Strategy for the establishment of *G9a* KO iMEF expressing hGem(1/110)-G9a-mVenus and hCdt1(1/100)-G9a-mVenus (Fig. 3a). (**b**) The established cell lines were stained by Hoechst 33342 and analyzed using a FACSAria SORP apparatus. Black line: total cells, blue line: AmCyan (+) cells, red line: mCherry (+) cells and green line: mVenus (+). (**c**) The established cell lines were analyzed for live cell imaging for fluorescent signals. The images were excerpted during the first 24 h. mVenus (upper panels), and AmCyan and mCherry (lower panels) are shown in combination using bright field microscopy. They were photographed every 30 min. (**d**) Fluorescence intensities are indicated in the line graphs. Blue line: AmCyan, red line: mCherry, and green line: mVenus. (**e**) The expression of G9a-mVenus protein was determined by western blot. mCherry and AmCyan were also determined to confirm the sorting specificity. (−) Total cells, A: AmCyan (+) sorted cells, and C: mCherry (+) sorted cells.

Using these G9a cell cycle-regulating expression constructs, *G9a* KO iMEFs were again transfected with tFucci(SCA)2.1 first and selected with puromycin (Fig. 4a). The puromycin-selected cells were further transfected with hGem(1/110)-G9a-mVenus or hCdt1(1/100)-G9a-mVenus. After blasticidin selection, AmCyan/mVenus double-positive but mCherry negative cells, and mCherry/mVenus double-positive but AmCyan negative cells were sorted to retrieve the cells transfected with hGem(1/110)-G9a-mVenus and hCdt1(1/100)-G9a-mVenus, respectively (Fig. 4a). FACS analysis confirmed that the mVenus signal was stably detected throughout the cell cycle in the cells transfected with G9a-mVenus, while the signal was highly restricted to mCherry-positive cells in hCdt1(1/100)-G9a-mVenus-transfected cells (Fig. 4b right panels). On the other hand, mVenus signals in hGem(1/110)-G9a-mVenus-transfected cells were detected from middle of the G1 phase to the M phase, as seen in Fig. 4b (2^nd^ bottom right panel). We further performed live fluorescent imaging and quantified the intensity of fluorescence signals using MetaMorph (Fig. 4c,d). The mVenus signals for hCdt1(1/100)-G9a-mVenus appeared and disappeared at almost the same time as the mCherry signals (Fig. 4d bottom right panel). On the other hand, the mVenus signals for hGem(1/110)-G9a-mVenus appeared before the shut-down of the mCherry signals (Fig. 4d upper right panel), and were detected until the end of the M phase when AmCyan signals disappeared (Fig. 4d upper middle panel). Furthermore, we examined the expression and quantity of the G9a-mVenus fusion protein with or without degron in the cells sorted for G1 and out-of-G1 phase, by western blot analysis. The expected molecular weight of G9a-mVenus fusion was detected in all the transfected cells (Fig. 4e). Also as expected, hCdt1(1/100)-G9a-mVenus was strongly enriched in the G1 (mCherry-positive) cell populations, although the level of expression was lower than that of endogenous G9a (Fig. 4e left upper panel, WT vs hCdt1(1/100)-G9a-mVenus lanes). hGem(1/110)-G9a-mVenus in the transfected cells was also enriched in out-of-G1 (AmCyan positive) cell populations, suggesting that hGeminin(1/110) or hCdt1(1/100) fusion G9a-mVenus molecules were degraded in the expected specific phase of the cell cycle. The data indicated the successful establishment of cell lines expressing G9a restricted to the G1 or late G1-M cell cycle phase.

### Recovery of H3K9me1 and me2 in cells expressing G9a restricted to G1 or late G1-M cell cycle phase

**Figure 5:**
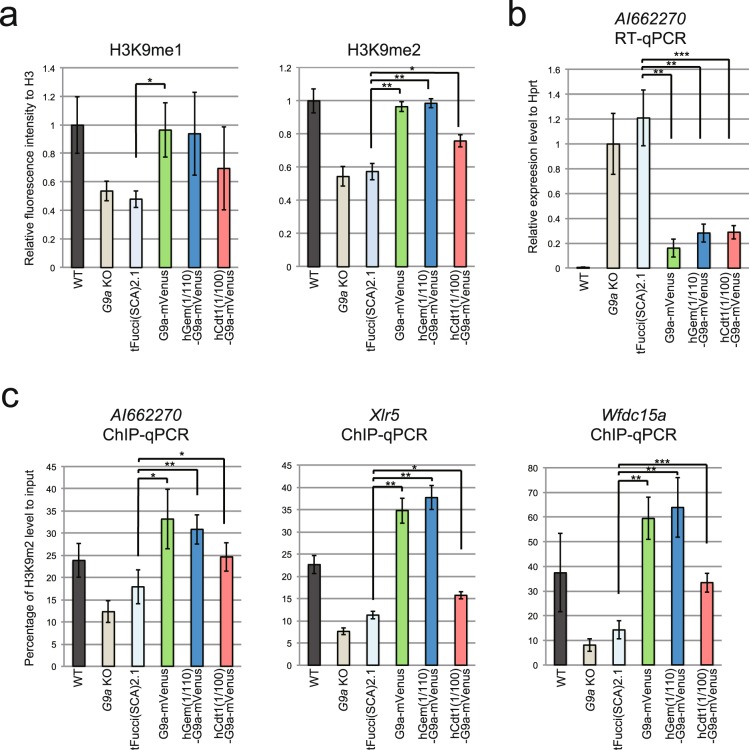
Profiling of G9a function in the cell cycle. (**a**) H3K9me1 and me2 levels were determined by western blot analysis using Odyssey CLs. The means of relative fluorescence intensity to H3 are shown in the graphs. me1: N = 3, me2: N = 4, independent experiments. Error bars indicate ± SD. *p < 0.05, **p < 0.01 and ***p < 0.001 by Student’s t-test. Compared to WT, *G9a* KO and tFucci(SCA)2.1 showed statistically significant differences (p < 0.05) for both H3K9me1 and me2. Original images are shown in (Fig. S3). (**b**) G9a target gene, *AI662270* mRNA level was measured by quantitative RT-PCR. The mean values show the relative expression level to *Hprt*. N = 4, independent experiments. Compared to WT, all other samples showed statistically significant differences (p < 0.05). (**c**) ChIP analysis for H3K9me2. Upstream of *AI662270, Xlr5 and Wfdc15a* were measured by quantitative PCR. *AI662270*: N = 5, *Xlr5* and *Wfdc15a*:N = 4 independent experiments. The mean values show percentage of H3K9me2 level to input. Compared to WT, *G9a* KO and hGem(1/110)-G9a-mVenus for *AI662270, G9a* KO and tFucci(SCA)2.1 for *Xlr5* and *Wfdc15a* showed statistically significant differences (p < 0.05).

We investigated H3K9me1, me2, and me3 levels in the established cell lines. H3K9me1 and me2 were decreased by approximately half in *G9a* KO iMEFs (Figs 5a and S3), but H3K9me3 was not significantly changed (Figs S1b and S5). The diminished level of H3K9me1 and me2 were completely recovered by G9a-mVenus expression (Figs 5a and S3). The diminished H3K9me1 tended to increase in the established cell cycle-specific G9a expressing cell lines (Figs 5a and S3). When hGem(1/110)-G9a-mVenus was expressed in *G9a* KO iMEFs, H3K9me2 was completely recovered to the wild type (WT) level as it was rescued by G9a-mVenus (Figs 2f, 5a and S3). H3K9me2 was also significantly increased in the cells expressing hCdt1(1/100)-G9a-mVenus, but the level of recovery was incomplete.

G9a inhibits expression of the target genes in a H3K9 methyltransferase dependent manner^7,8^. Among the known G9a target genes, such as *Magea*, which are derepressed in *G9a* KO embryonic stem cells (ESCs)^7,8^, we could not identify a gene that was also silenced in iMEFs by G9a. Therefore, we changed to a different approach to identify such gene(s). Jmjd1a and 1b (also known as Jhdm2a and 2b or Kdm3a and 3b) are lysine demethylases for H3K9me1 and me2^15^. We looked for genes that have been reported to be down-regulated in *Jmjd1a* or *1b* KO and *Jmjd1a, 1b* double KO ESCs^16^. We then examined their expression level in WT and *G9a* KO ESCs and iMEFs, and discovered a new G9a target gene, *AI662270*, which was commonly up-regulated in *G9a* KO ESCs and iMEFs (Fig. S4). *AI662270* was de-repressed in *G9a* KO iMEFs and was re-suppressed by introduction of G9a-mVenus (Fig. 5b). *AI662270* expression was also suppressed in the *G9a* KO iMEFs expressing both hGem(1/110)-G9a-mVenus and hCdt1(1/100)-G9a-mVenus (Fig. 5b). Furthermore, the level of H3K9me2 upstream of *AI662270* was significantly decreased in *G9a* KO iMEFs and recovered in the cell lines that expressed all the constructs (Fig. 5c). We also examined *Xlr5* and *Wfdc15a*, which are other G9a target genes that are repressed in *G9a* KO ESCs^8^, but not in iMEFs (Fig. S1c). H3K9me2 ChIP-qPCR analysis revealed the decreased level of H3K9me2 upstream of *Xlr5* and *Wfdc15a* in G9a KO iMEFs (Fig. 5c). The H3K9me2 level was recovered in the cell lines expressing all the constructs (Fig. 5c). From those results, we conclude that G9a can induce H3K9me2 in the G1 phase of the cell cycle.

## Discussion

The Fucci system allows the monitoring of the cell cycle in cell culture and during embryonic and tissue development^12,17–19^. The cell cycle-specific degron employed in the Fucci system has also been applied for other purposes, including the out-of-G1-specific Cas9 expression by conjugation of hGeminin(1/110) to Cas9, for cell cycle regulated CRISPR/Cas9-mediated genome editing^20,21^, and for cell cycle-specific expression of DNA cytosine deaminase (also termed activation induced deaminase (AID))^22^. However, the cell cycle-specific expression of epigenome regulating factors using such a degron system has not been described. We applied this degron system to G9a and achieved in making cells that were able to express G9a only during G1 or late G1-M cell cycle phases.

We conjugated hGeminin(1/110) to either the N- or C-terminus of G9a (G9a-mVenus or mVenus-G9a, respectively). N-terminal conjugation resulted in the immediate depletion upon entering G1 phase (Fig. 3c). Since G9a forms a complex with partner molecules GLP and Wiz via its SET domain, which is in the C-terminal region of G9a^23,24^, ubiquitination of G9a by E3 ubiquitin ligase or degradation of the ubiquitinated fusion protein by proteasome may be prevented by the tertiary complex formation in mVenus-G9a-hGem(1/110) and mVenus-G9a-F-hGem(1/110). Although signaling molecules are quite different between hGeminin- and hCdt1-degron-mediated protein degradation, it is still possible that induction of G9a degradation induced by the hCdt1(1/100)-degron might be suppressed if it is conjugated to the C-terminus of G9a. Therefore, we conjugated the hCdt1(1/100)-degron to the N-terminus of G9a and observed that it also worked well for out-of-G1-specific degradation. In the Fucci system, both Geminin and Cdt1 degrons are conjugated to the C-termini of two fluorescent proteins and induce efficient degradation of fusion partners in the G1 phase and out-of-G1 phase of the cell cycle, respectively. Thus, it is important for researchers to examine whether N-or C-terminal conjugation of Geminin/Cdt1 degron works for efficient degradation of the target protein in the expected cell cycle stage. Lastly, even though hGem(1/110)-G9a-mVenus can be depleted upon entering the G1 phase, its expression is induced in the middle of the G1 phase. Further experiments are necessary concerning complete regulation of out-of-G1 specific G9a expression using this system.

G9a and GLP prefer core-histone, but not nucleosomal histone, as the *in vitro* methylation reaction substrate^9^. However, neighboring nucleosomes containing H3K9me1 or me2 are able to stimulate methylation catalysis using the nucleosomal histone substrate^10^. This suggests that parental nucleosomes containing H3K9me1 or me2 may be crucial for recovery of G9a/GLP-mediated H3K9 methylation on newly generated and incorporated nucleosomes after cell division. The level of H3K9me2 recovers relatively quickly from the S to G2/M phases after cell division^6^. It is conceivable that this recovery is mediated by G9a, since G9a is the typically major enzyme for H3K9me2. However, there is not yet direct proof for this. Furthermore, it is unclear whether H3K9me2 is also catalyzed in the G1 phase. Presently, using cells that expressed G9a only during the G1 or out-of-early G1 phase, we found that G9a can induce H3K9me2 in not only S/G2/M phases but also in G1 phase of the cell cycle. These findings are crucial for the development of disease treatment approaches based on epigenome-manipulation. The demonstration of H3K9me2 recovery specific in G1 is especially important, because the long-lived, terminally-differentiated cells in the human body, such as neurons, are mostly post-mitotic G0-G1 cell populations.

## Materials and Methods

### Construction of Fucci vectors

tFucci(SCA)2.1 was modified from tFucci(SA)2.2^14^. hCdt1(30/120) was replaced with hCdt1(1/100) and mTurquoise was replaced with AmCyan. It was introduced into pPBCAG-BstXI-IP^25^. mVenus-G9a-hGem(1/110), mVenus-G9a-F-hGem(1/110), G9a-mVenus, hGem(1/110)-G9a-mVenus, and hCdt1(1/100)-G9a-mVenus were introduced into pPBCAG-cHA-IB^25^.

### Establishment of G9a KO iMEFs

All animal experiments were performed under the animal ethical guidelines of Kyoto University (experiment number A12-6-2, approved by Animal Experimentation Committee of Kyoto University). E13.5 MEFs were obtained from the breeding of *G9a*^*flox/flox*^ ^26^; *Esr1-Cre*^+^ male mice with *G9a*^*flox/flox*^ female mice (*Esr1-Cre*: B6N.129S6(Cg)-*Esr1*^*tm1.1(cre)And*^/J), with transformation by SV40 T antigen^27^. *G9a* KO iMEFs were established by 4-OHT treatment (Fig. S1a).

### Cell culture and establishment of cell lines

*G9a* KO iMEFs were cultured in Dulbecco’s modified Eagle’s medium (DMEM, 08458-16, Nacalai Tesque) containing 10% fetal calf serum (FCS), MEM non-essential amino acids (Gibco), and 100 μM 2-mercaptoethanol. Approximately 5 × 10^4^–10^5^ *G9a* iMEFs were passaged in one well of 6-well plate and cultured for ~4 h to overnight. Two micrograms of tFucci(SCA)2.1 vector was transfected with 1 µg pCAG-PBase vector^28^ into cells using FuGENE HD (Promega). After overnight incubation, transfected cells were passaged in 10-cm culture dishes. The next day, drug selection with the culture medium containing 2 µg/ml puromycin (InvivoGen) was started. The cells were passaged several times and the drug selection was continued for approximately 5–7 days. In addition, G9a-mVenus vectors were transfected into tFucci(SCA)2.1 iMEFs as described above. Transfected cells were selected using culture medium containing 10 µg/ml blasticidin (InvivoGen). They were also passaged several times and the drug selection was continued for approximately 5–7 days. After drug selection, these cells were sorted to isolate AmCyan positive cells for tFucci(SCA)2.1, AmCyan and mVenus double-positive cells for G9a-mVenus and hGem(1/110)-G9a-mVenus, and mCherry and mVenus double-positive cells for hCdt1(1/100)-G9a-mVenus using a FACSAria SORP apparatus (BD Bioscience).

### Flow cytometry

The cells were harvested and stained using culture medium containing 10 µg/ml Hoechst 33342 (Dojindo) for 30 min at 37 °C in a incubator with 5% CO_2_. It was mixed every 10 min. After incubation, the staining solution was removed and the cells were suspended in culture medium, followed by nylon-mesh filtration and FACSAria SORP examination. The data were analyzed using FlowJo software (BD Bioscience).

### Live cell imaging

Passage of 10^5^ cells was carried out in 35-mm glass base dish (Iwaki). After overnight culture, the cells were washed three times and cultured with DMEM (31053028, Gibco) containing 0.1% FCS, MEM non-essential amino acids (Gibco), glutamine (Gibco), sodium pyruvate (Gibco), and 100 μM 2-mercaptoethanol. Images were taken every 30 min for 72 h using a model LCV110 microscopy (Olympus). The images were analyzed using MetaMorph software (Molecular Devices).

### Western blot analysis

For the detection of G9a, mVenus, mCherry, and AmCyan, cells were sorted for AmCyan or mCherry-positive cells using the FACSAria SORP, washed with phosphate buffered saline, and lysed with 420 mM NaCl lysis buffer (420 mM NaCl, 20 mM HEPES-KOH pH 7.5, 1.5 mM MgCl_2_, 0.1% NP40, and protease inhibitor cocktail 25955-11; Nacalai Tesque). Anti-G9a antibody (#8620, Perseus Proteomics)^23^, anti-GLP antibody (#0422, Perseus Proteomics)^23^, anti-Tubulin antibody (T5168, Sigma Aldrich), anti-green fluorescent protein (GFP) antibody (598, MBL), anti-red fluorescent protein (RFP) antibody (PM005, MBL), and anti-reef coral fluorescent protein (RCFP) antibody (632475, Clontech) were used as primary antibodies. Enhanced chemiluminescence (ECL) anti-mouse IgG HRP antibody (NA931, GE Healthcare) and ECL anti-rabbit IgG horseradish peroxidase (HRP) antibody (NA934, GE Healthcare) were used as secondary antibodies. Western Lightning Plus ECL (PerkinElmer) was used to detect the signal. For quantitative measurement of H3, H3K9me1, H3K9me2 and H3K9me3, cells were harvested, washed with PBS, and acid-extracted as previously described^7^. Anti-H3 antibody (07–690, Merck Millipore), anti-H3K9me1 antibody (2F7a, CMA306)^29^, anti-H3K9me2 antibody (6D11, CMA317)^30^, and H3K9me3(2F3, CMA318)^30^ were used as primary antibodies. IRDye 800CW goat anti-mouse (926–32210, LI-COR) and IRDye 680RD goat anti-rabbit (926–68071, LI-COR) were used as second antibodies. The fluorescent intensities were detected using an Odyssey CLx device (LI-COR). The data were analyzed by Image Studio Lite software (LI-COR).

### Quantitative RT-PCR analysis

Total RNA was purified using an RNeasy Plus Mini Kit (QIAGEN). cDNAs were synthesized using the Omniscript Reverse Transcription Kit (QIAGEN), Random Primer (TaKaRa Bio), and RNase inhibitor (TOYOBO). *AI662270, Magea, Xlr5*, and *Wfdc15a* mRNA expression were measured using the Power SYBR Green PCR Master Mix (Applied Biosystems) in the StepOnePlus system (Applied Biosystems). The primer sets used were: 5′-CCAGGAGGAAAACCAAATAGCC-3′ (forward) and 5′-AAGACGGTGAGCAGAGCAAAAC-3′ (reverse) for *AI662270*, 5′-ATTTCCCTTCTAACCCCAGGTC-3′ (forward) and 5′-TCCTCAAGTCACCCAAACAGAG-3′ (reverse) for *Magea*, 5′-TAGTAGCCAGTGCAGCTCCTTTCTC-3′ (forward) and 5′-CACAGAGCTGCTGGGAGTTCACTTA-3′ (reverse) for *Xlr5*, 5′-TCTTCTTGACTGCCCGTTTG-3′ (forward) and 5′-TTCCACACACCGCATCTGAC-3′ (reverse) for *Wfdc15a*, and 5′-CAGGCCAGACTTTGTTGGAT-3′ (forward) and 5′-TTGCGCTCATCTTAGGCTT-3′ (reverse) for *Hprt*^31^ as an internal control.

### Chromatin Immunoprecipitation (ChIP)

ChIP analysis was performed as described previously^8^ using anti-H3K9me2 antibody (6D11) and Dynabeads M-280 Sheep anti-mouse IgG (Thermo Fisher) were used. The StepOnePlus system was used for analysis using the following primer sets: 5′-TGAAGACCAAGGAAGTGAGAAGAC-3′ (forward) and 5′-AGTGGCTGTTGTGTGAAGGAAG-3′ (reverse) for *AI662270*, 5′-CGGAACCTCTGTGCTGACCTGTGGT-3′ (forward) and 5′-CGCTCCAGAACAAAATGGCGCAGA-3′ (reverse) for *Magea2*, 5′-GGCATCTCGAGACTTAAGGTAAAGC-3′ (forward) and 5′-CACCGAACCCCTCTTACTCTCCAGG for *Xlr5*, and 5′-GGATAACCAGCAGGGAAGCTGAGG-3′ (forward) and 5′-TCTCTAGGTGCCTGAACCTACAGC-3′ (reverse) for *Wfdc15a*^8^.

## Supplementary information

Supplementary Fig. 1-6
