# Supplementary material for: G9a-dependent histone methylation can be induced in G1 phase of cell cycle
Source: Sci Rep. 2019 Jan 30;9:956. doi: 10.1038/s41598-018-37507-5 (PMC6354049; doi:10.1038/s41598-018-37507-5)

Supplementary Figures

Title: G9a-dependent histone methylation can be induced in G1 phase of cell cycle

Mikiko Fukuda[1], Asako Sakaue-Sawano[2], Chikako Shimura[1], Makoto Tachibana[3, 4], Atsushi Miyawaki[2], Yoichi Shinkai[1§]

[1]Cellular Memory Laboratory, RIKEN Cluster for Pioneering Research, 2-1 Hirosawa, Wako, Saitama 351-0198, Japan

[2]Laboratory for Cell Function Dynamics, RIKEN Center for Brain Science, 2-1 Hirosawa, Wako, Saitama 351-0198, Japan

[3]Institute of Advanced Medical Sciences, Tokushima University, 3-18-15 Kuramoto-cho, Tokushima 770-8503, Japan

[4]Experimental Research Center for Infectious Diseases, Institute for Virus Research, Kyoto University, 53 Shogoin, Kawara-cho, Sakyo-ku, Kyoto 606-8597, Japan

[§]Corresponding author, Cellular Memory Laboratory, RIKEN Cluster for Pioneering Research, 2-1 Hirosawa, Wako, Saitama 351-0198, Japan; Tel.: +81 48-467-9370; Fax: +81 48-462-4670; E-mail: yshinkai@riken.jp

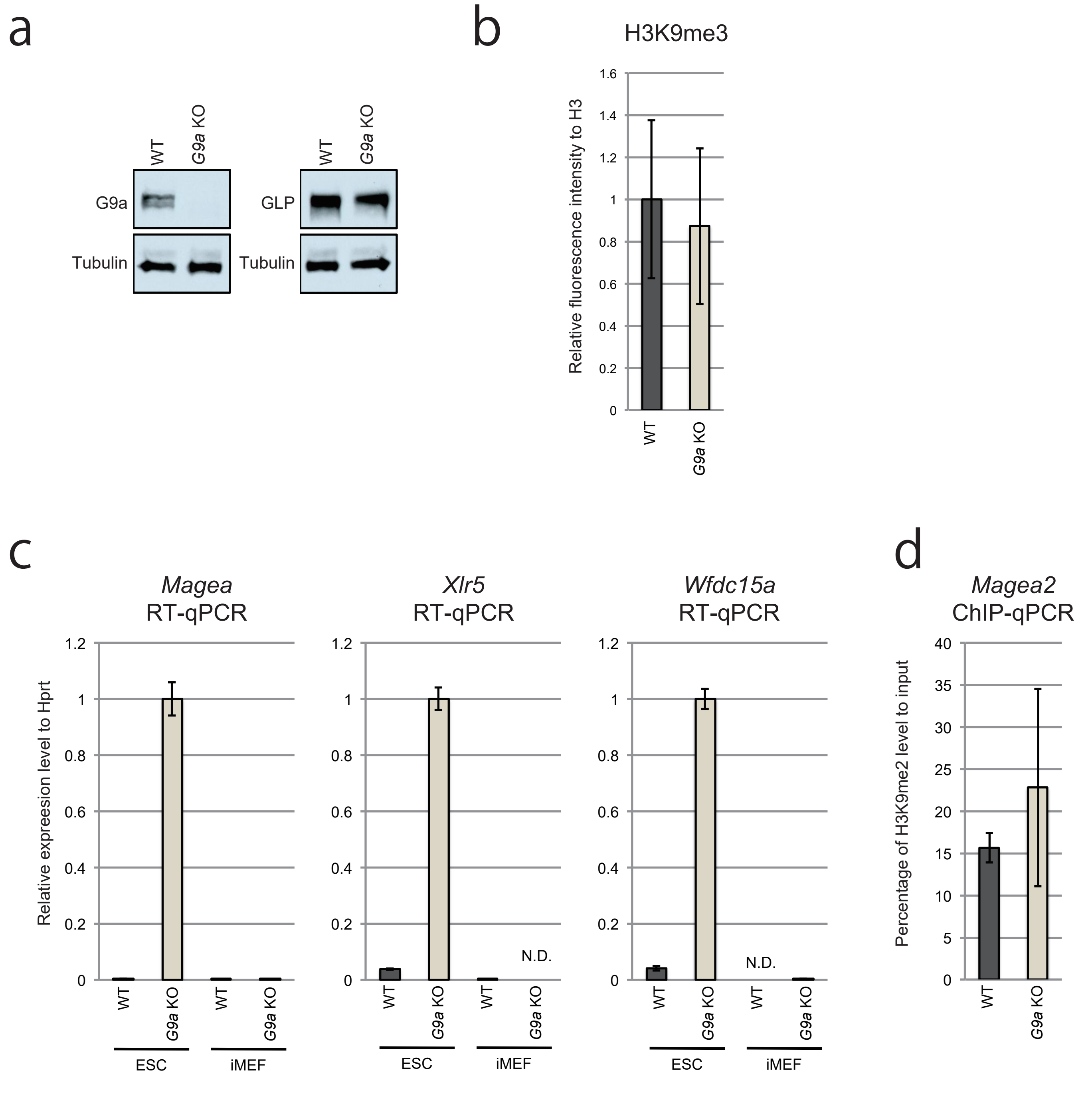

Supplementary Figure 1. Phenotypic analysis of *G9a* KO iMEFs.

a) Contents of G9a and GLP in *G9a* KO iMEFs were examined by western blot analysis. b) H3K9me3 level was determined by western blot using Odyssey CLs. N=5, independet experiments. c) Expression of G9a target genes, *Magea, Xlr5* and *Wfdc15a* were measured by quantitative RT-PCR. The mean values show the relative expression level to *Hprt.* N=3, technical replicates. N.D., not detected. d) Chip analysis for H3K9me2. Upstream of *Magea2* was measured by quantitative PCR. N=3, independet experiments. Error bars indicate ±SD.

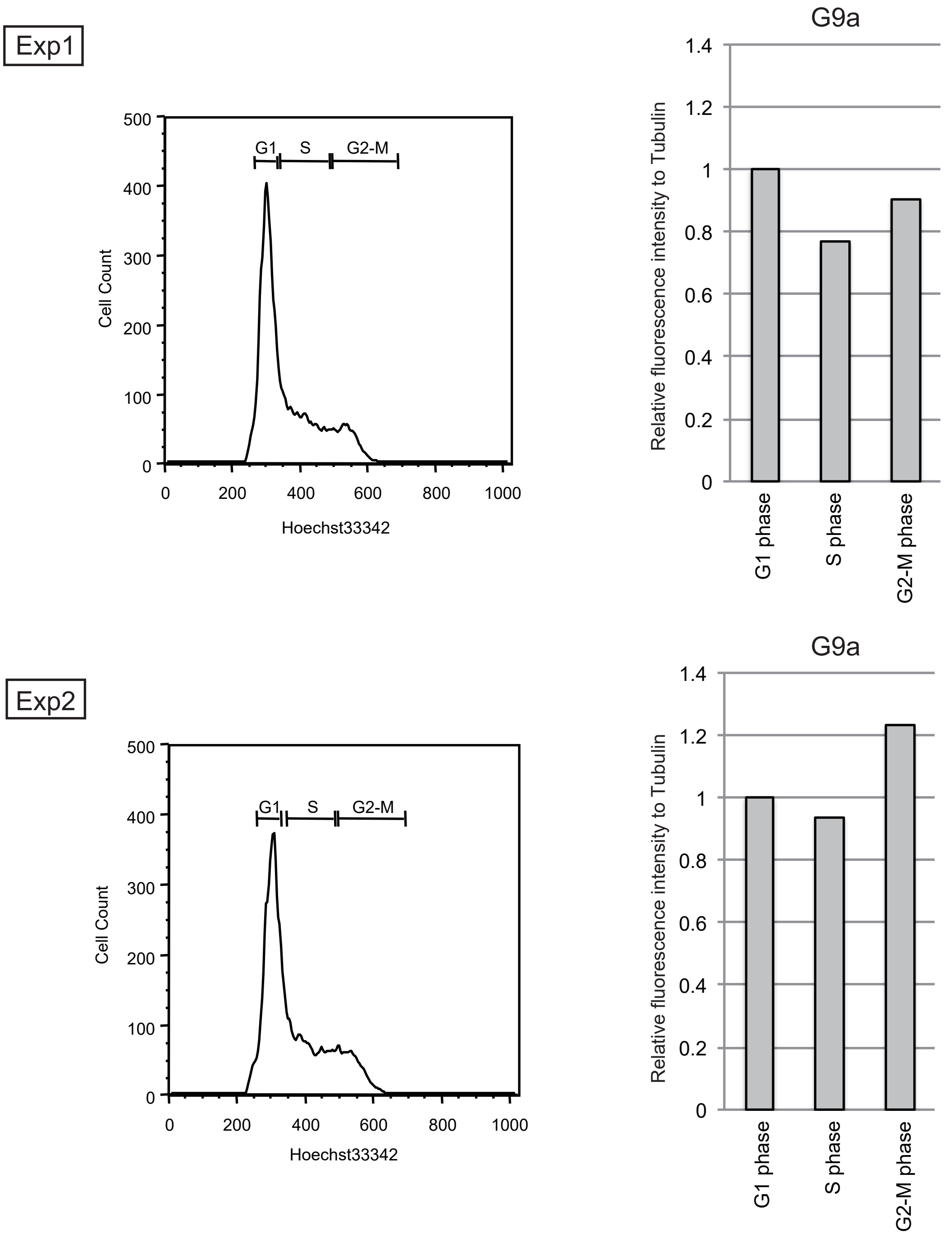

Supplementary Figure 2. The amount of G9a protein in the cell cycle.

The cells were stained by Hoechst33342 and sorted by FACSAria SORP (Left). G9a was detected by western blot analysis using Odyssey CLs. The relative fluorescence intensity of G9a to Tubulin is showed in the graphs (Right). Experiments were performed 2 times.

Fig.2f

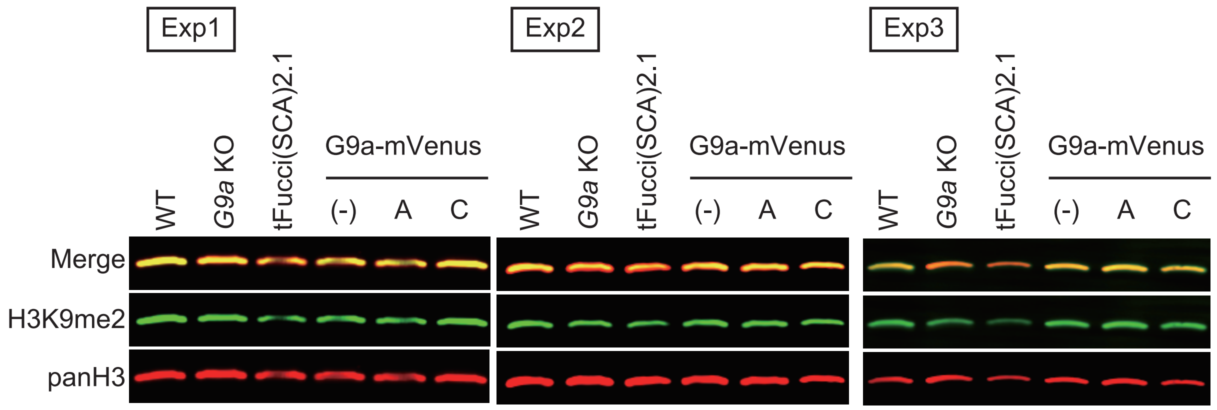

Fig.5a left

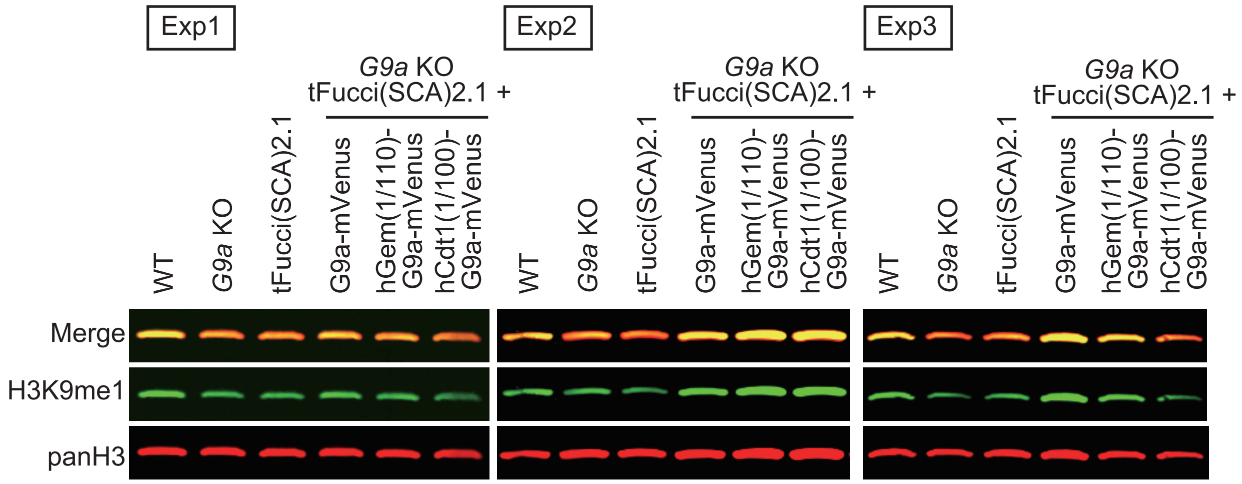

Fig.5a right

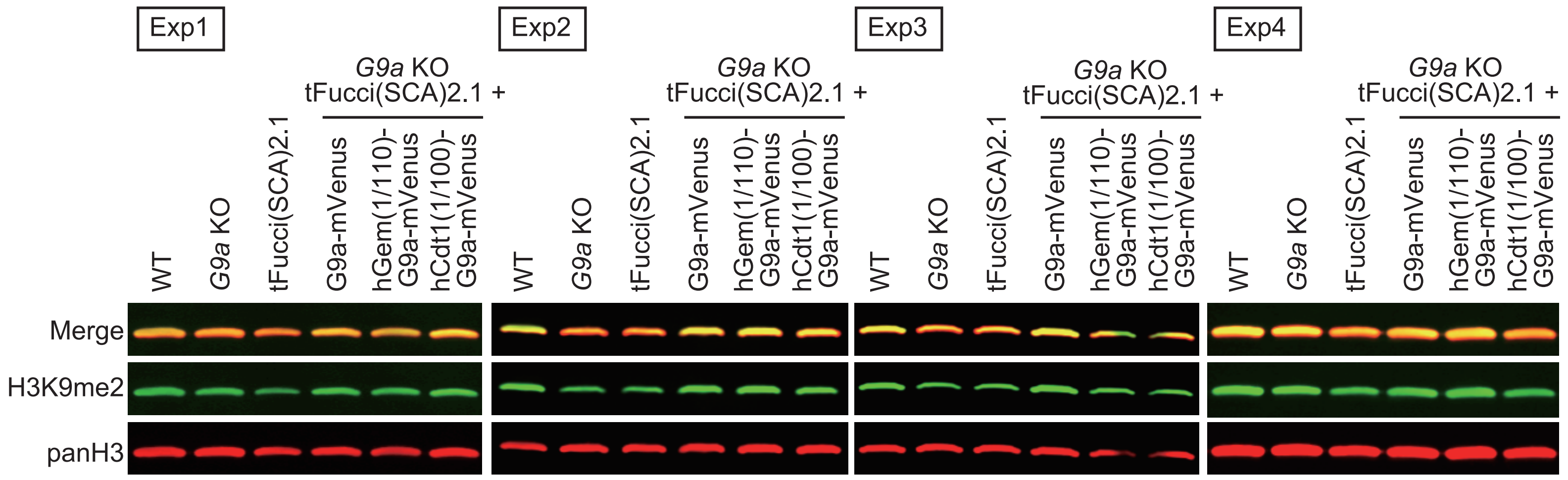

Supplementary Figure 3. Images of the immunoblotting in Fig.2f and Fig.5a.
Histones were immunoblotted by anti-H3K9me1 or me2 and anti-H3 antibodies as primary antibodies and detected by IRDye secondary antibodies. Three (Fig.2f and Fig.5a left) and four (Fig.5a right) independent experiments were performed. Fig.2f, (-): total cells, A: AmCyan (+) sorted cells, C: mCherry (+) sorted cells.

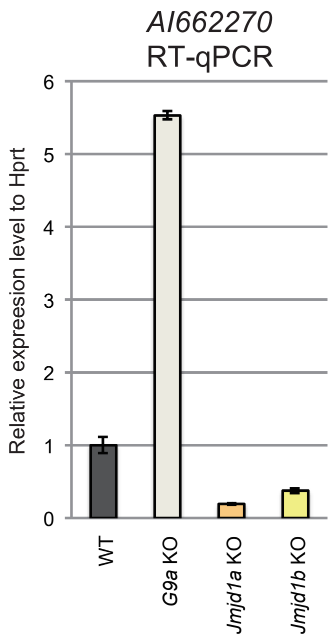

Supplementary Figure 4. AI662270 expression profile in WT and *G9a* KO ESCs.
AI662270 mRNA was mesuered by quantitative RT-PCR. WT: TT2, *G9a* KO: 22-10, *Jmjd1a* KO: 31-1[28] and *Jmjd1b* KO: D4-1[27]. All KO ES lines are derived from TT2. N=3, technical replicates. Error bars indicate ±SD.

Fig.S1b

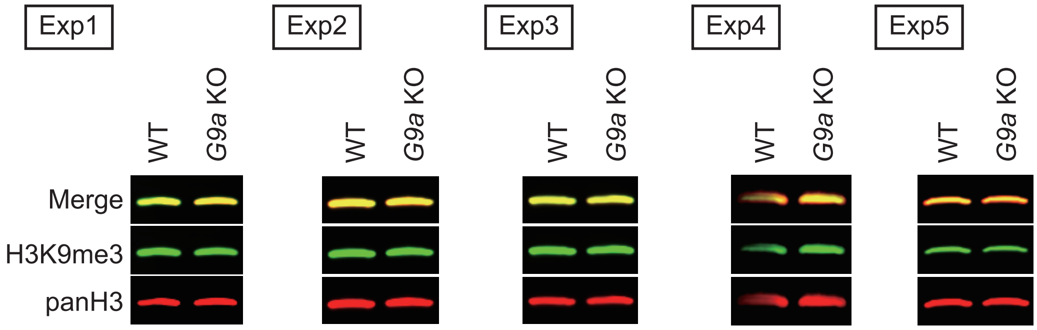

Fig.S2 right

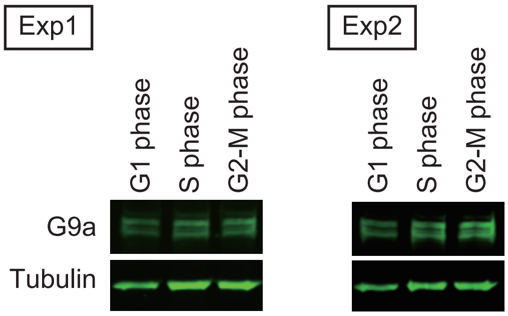

Supplementary Figure 5. Images of the immunoblotting used in Fig.S1b and Fig.S2. Histones were immunoblotted by anti-H3K9me3 and anti-H3 antibodies (Fig.S1b) and G9a and Tubulin were immunoblotted (Fig.S2) as primary antibodies and detected by IRDye secondary antibodies. Five (Fig.S1b) and two (Fig.S2) independent experiments were performed.

Fig.2e

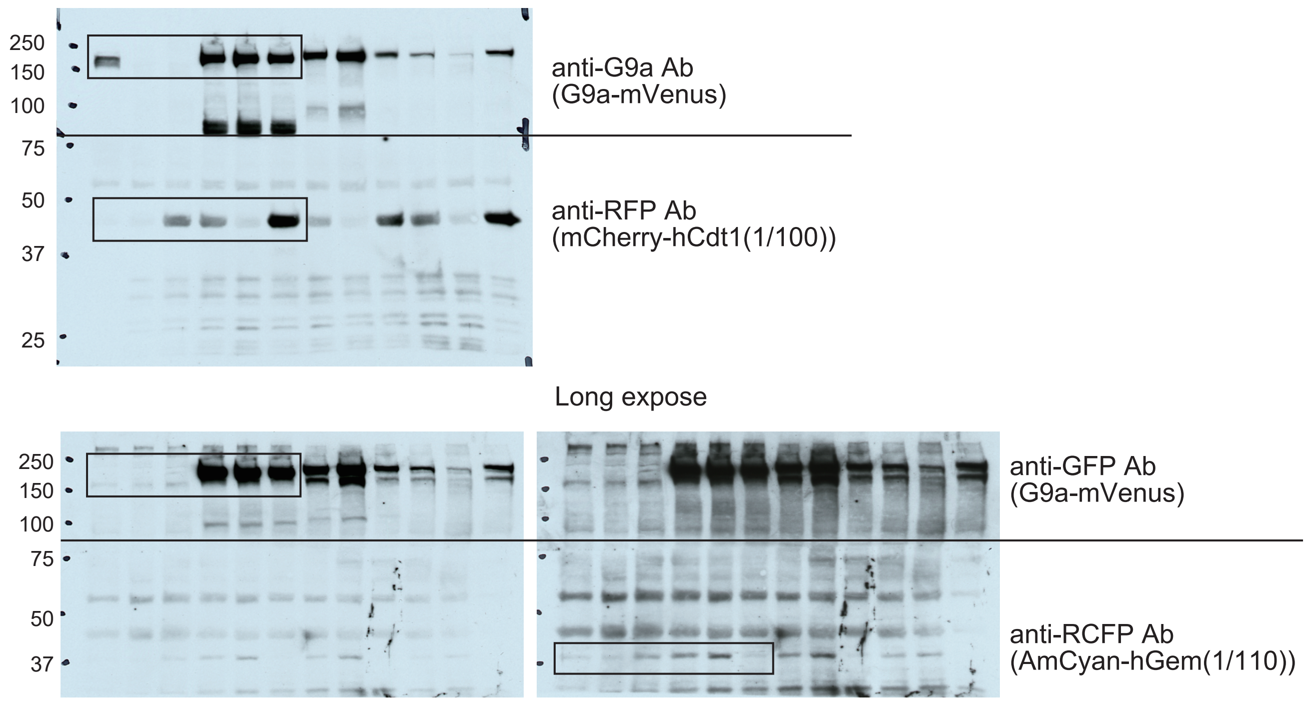

Fig.4e

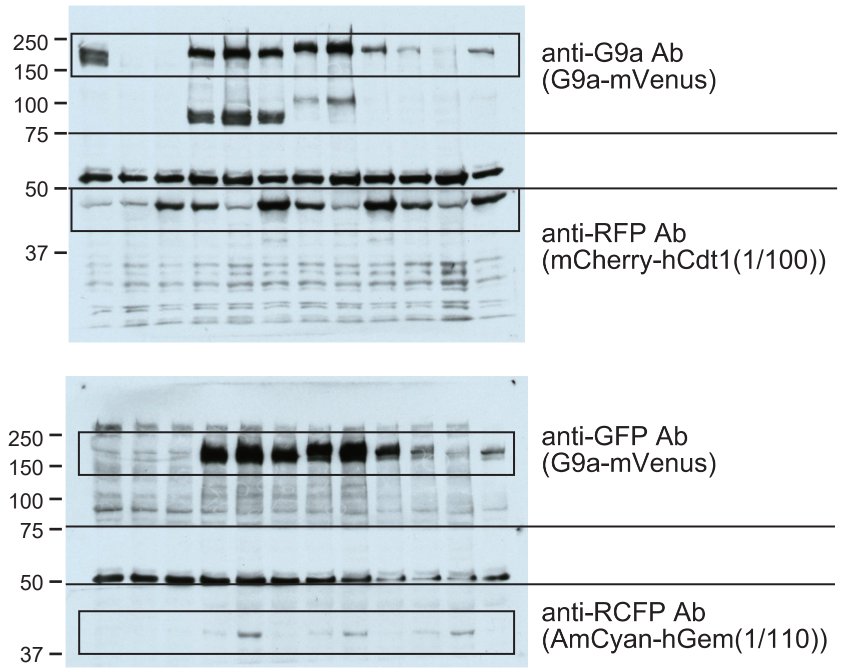

Fig.S1a

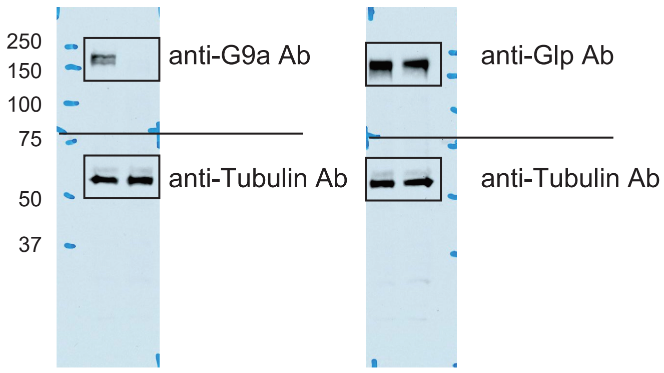

Supplementary Figure 6. Uncropped images of the immunoblotting in Fig.2e, Fig.4e, Fig.S1a, Fig.S3 and Fig.S5.

Each graph picked selection area.

Fig.S3 (Fig.2f)

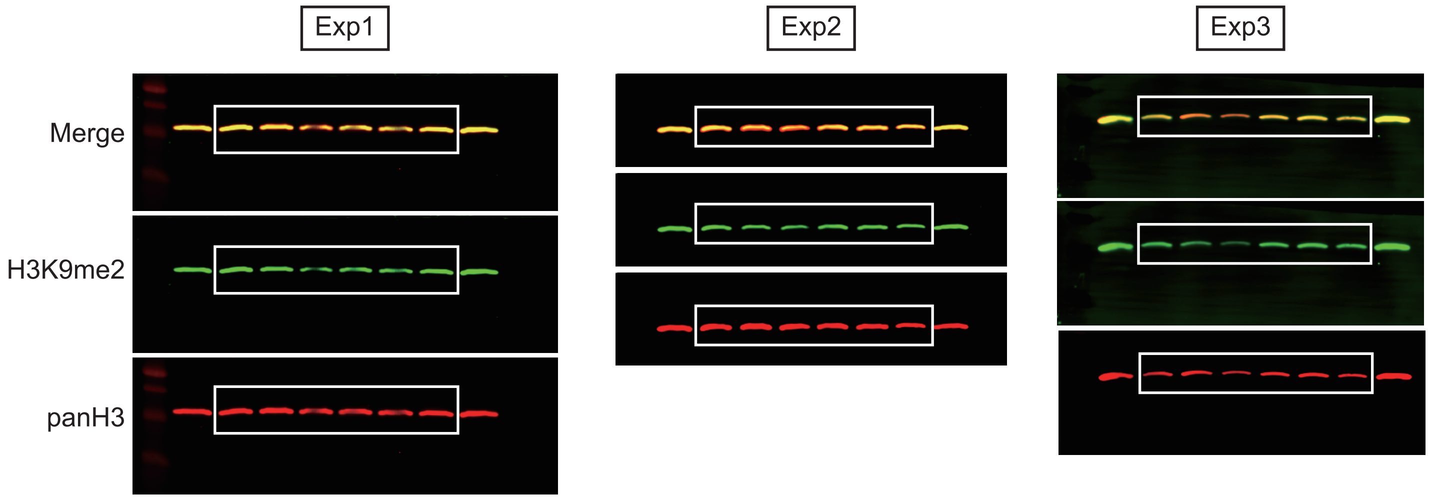

Fig.S3 (Fig.5a left)

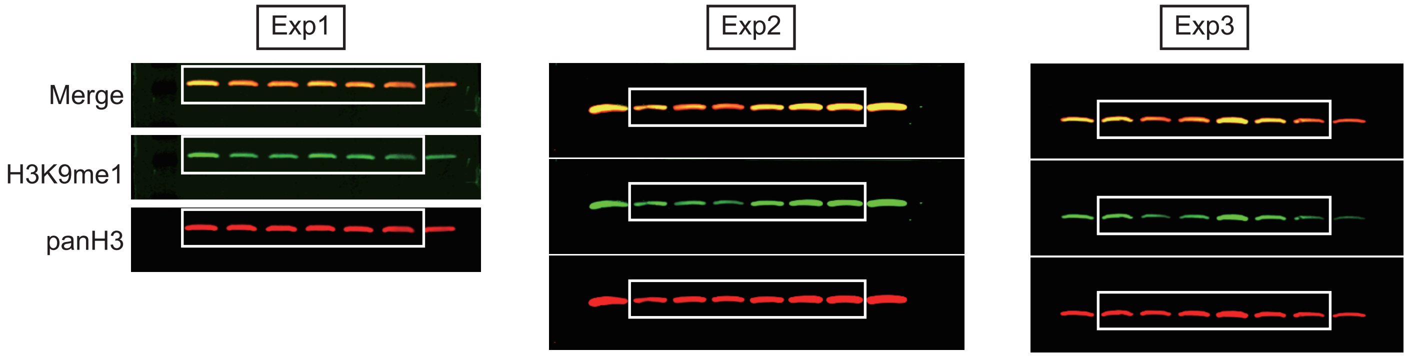

Fig.S3 (Fig.5a Right)

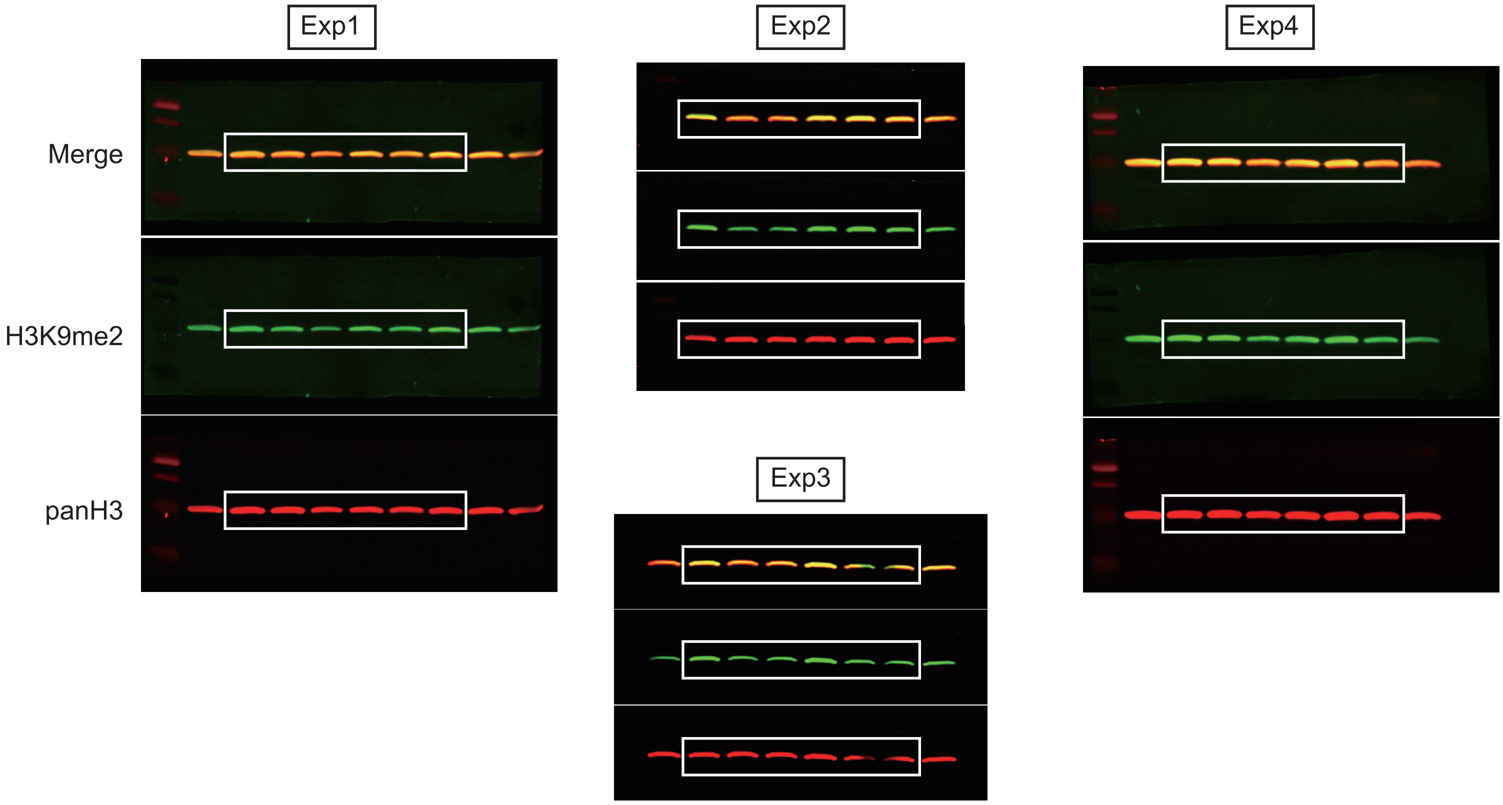

Fig.S5 (Fig.S1b)

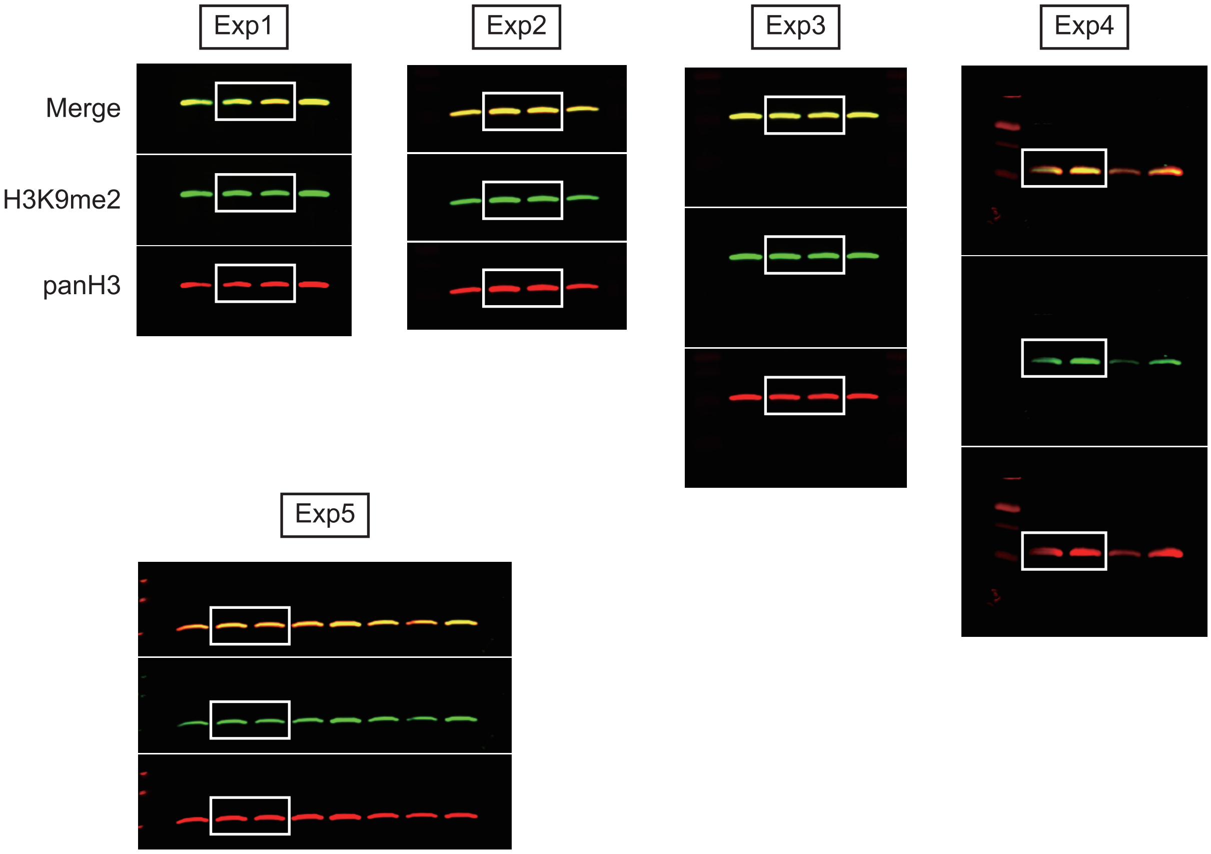

Fig.S5 (Fig.S2 Right)

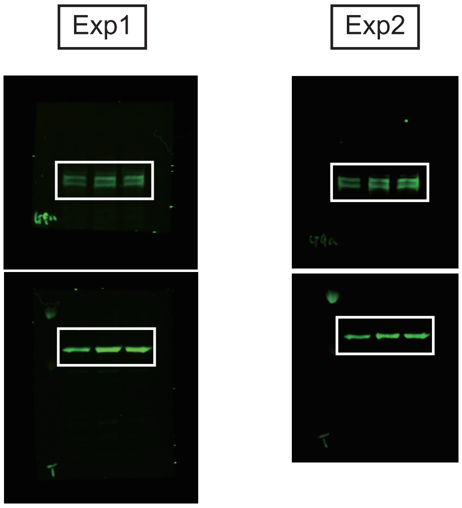

Supplement: Supplementary file 1 — Supplementary Fig. 1-6 [file 41598_2018_37507_MOESM1_ESM.pdf]
